# Molecular Characterization of NDL1-AGB1 Mediated Salt Stress Signaling: Further Exploration of the Role of *NDL1* Interacting Partners

**DOI:** 10.3390/cells10092261

**Published:** 2021-08-31

**Authors:** Nidhi Gupta, Abhishek Kanojia, Arpana Katiyar, Yashwanti Mudgil

**Affiliations:** Department of Botany, University of Delhi, New Delhi 110007, India; nidhigupta.v@gmail.com (N.G.); abhishek.ak0447@gmail.com (A.K.); katiyar.arpana@gmail.com (A.K.)

**Keywords:** NDL1, AGB1, ANNAT1, SLT1, NaCl, *Arabidopsis*, abiotic stress

## Abstract

Salt stress is considered to be the most severe abiotic stress. High soil salinity leads to osmotic and ionic toxicity, resulting in reduced plant growth and crop production. The role of G-proteins during salt stresses is well established. AGB1, a G-protein subunit, not only plays an important role during regulation of Na^+^ fluxes in roots, but is also involved in the translocation of Na^+^ from roots to shoots. N-Myc Downregulated like 1 (NDL1) is an interacting partner of G protein βγ subunits and C-4 domain of RGS1 in *Arabidopsis*. Our recent in-planta expression analysis of *NDL1* reported changes in patterns during salt stress. Based on these expression profiles, we have carried out functional characterization of the AGB1-NDL1 module during salinity stress. Using various available mutant and overexpression lines of *NDL1* and *AGB1*, we found that *NDL1* acts as a negative regulator during salt stress response at the seedling stage, an opposite response to that of *AGB1*. On the other hand, during the germination phase of the plant, this role is reversed, indicating developmental and tissue specific regulation. To elucidate the mechanism of the AGB1-NDL1 module, we investigated the possible role of the three NDL1 stress specific interactors, namely ANNAT1, SLT1, and IDH-V, using yeast as a model. The present study revealed that NDL1 acts as a modulator of salt stress response, wherein it can have both positive as well as negative functions during salinity stress. Our findings suggest that the *NDL1* mediated stress response depends on its developmental stage-specific expression patterns as well as the differential presence and interaction of the stress-specific interactors.

## 1. Introduction

Soil salinity is one of the major abiotic stresses that affect plant growth, development, and productivity. Understanding Na^+^ sensing and signaling is important for development of salt (NaCl) tolerant crops. The phenomenon of salt stress tolerance has been studied throughout the years, yet the molecular understanding remains imprecise. One of the favorably discussed pathways during salt stress signaling is the salt overlay sensitive (SOS) pathway [1]. During salt stress, transient increase of Ca^2+^ is sensed via SOS3, a cytosolic Ca^2+^ binding protein [2]. SOS3 interacts and activates SOS2 forming SOS2/SOS3 kinase complex [3]. Phosphorylation of SOS2/SOS3 kinase complex is followed by activation of SOS1 (Na^+^/H^+^) antiporter that reinstates ionic homeostasis [4]. The role of plasma membrane during salt stress signaling is still elusive [5,6].

G protein signaling is extensively studied in plasma membrane signaling pathway. In *Arabidopsis* the G-protein complex comprises of three components, i.e., a Gα subunit, various isoforms of Gβγ dimer, and a 7-transmembrane (7-TM) regulator of G signaling (RGS) protein [7,8]. Mutant analyses of the various G-protein subunits across spermatophyte lineages suggest a conserved stress-related role of G-proteins [9]. In *Arabidopsis* null-mutants analyses of *AGB1*, triple *XLG* and triple *AGG* displayed smaller and chlorotic leaves in comparison to wild type, when grown on medium supplemented with NaCl [10,11,12]. However, the *gpa1* and *rgs1* mutants displayed contrastingly larger and less chlorotic leaves after NaCl treatments [11]. In rice and maize, null mutant analysis of Gα subunit at high salt concentration contributes to the attenuation of leaf senescence, cytoplasm electrolyte leakage, and chlorophyll degradation [13], though the overexpression of *RGG1* in rice contributes towards improved salt tolerance without affecting yield [14].

In animal systems, various G protein downstream interactors molecules have been investigated during stress responses. However in plant systems very few G protein interactors have been well characterized [15,16,17,18]. In 2011, the G protein interactome was elucidated, where a sum of 544 interactions between 433 proteins was established through the yeast two-hybrid interaction method [19]. Therein, NDL1 was also used as bait in the G protein interactome and was found to have 62 interacting partners. Out of these, 73% (45 out of 62) of the interactors have either predicted/or established roles in diverse biotic and abiotic stress responses [20].

Plant NDR proteins were reported first time as a transmitting tissue expressed protein in sunflower [21]. In *Arabidopsis*, the *NDL* gene family consists of three members, *NDL1*, *NDL2*, and *NDL3*, respectively, they share 75% identity at the protein level [17]. Animal homologs of *NDL* gene family have been found to be involved in various stress responses, such as hypoxia, DNA damage, presence of reducing agents and metal ions like nickel, cobalt, and iron, and in response to increased Ca^2+^ [22,23,24,25]. A recent study about the correlation of the gene expression and morpho-physiological traits during water deficient conditions has indicated *NDL1* as a biomarker under such conditions. The expression of *NDL1* positively correlated with the rate of transpiration and projected rosette area under water deficient conditions [26], suggesting involvement of *NDL1* during osmotic stress responses.

Our present study focuses on role of NDL1-AGB1 during salt stress responses and its association with putative interactors using yeast as model. Mutant analysis of *ndl1-2* and native overexpression analysis of *NDL1* in both Col-0 and *agb1-2* backgrounds have shown the involvement of *NDL1* in salinity stress during different stages of plant development. We analyzed *ndl1-2* mutant wherein we observed reduced germination under salt stress, demonstrating that *NDL* aids rescue from salinity stress during early stages of plant development. Contrary to germination observation, we found enhanced rosette formation of *ndl1-2* and *rgs1-2* mutant, a phenotype opposite to *agb1-2* and *NDL-GUSagb1-2* mutants which showed a reduced rosette area as compared to Col-0 and *NDL-GUS*. This latter observation therefore indicates that *NDL1* negatively regulates salinity induced stress response. Together, our study suggests a clear dichotomy in *NDL1* function during salinity induced stress response wherein it aids in withstanding salt stress during the early germinative stage but switches to negatively impacting stress response at vegetative stage. In-silico analysis shows increase in *NDL1* expression under salt stress, while the expression of *AGB1* does not undergo much variation. However, Ming et.al in 2015 [27] demonstrated that *AGB1* is necessary for normal growth under salt stress. We tested the potentially dual and opposite roles of *NDL1* in yeast. Here, *SLT1* showed increased growth with *NDL1* while *ANNAT1* showed decreased growth with *NDL1*. This explains that, during salt stress response, *NDL1* exerts it dual and opposite function by associating with putative interactors. We transformed *NDL1* with *IDH-V* and found it has no effects. This demonstrates the specificity of *NDL1* directed phenotype with its potential interactors.

## 2. Material and Methods

### 2.1. In-Silico Analysis

Abiological general repository for interaction datasets, https://thebiogrid.org/21021/summary/Arabidopsis-thaliana/ndl1.html (accessed on 20 March 2020) [28], was used to retrieve the information about different interactors of NDL1. The interactors were searched for their involvement in salt stress using various online tools and available literature. Micro-array data for *NDL1* and putative interactors during salt stress were retrieved from TAIR electronic Fluorescent Pictograph (eFP) browser http://bar.utoronto.ca/efp/cgi-bin/efpWeb.cgi (accessed on 22 March 2020) [29], in the form of fold change values at different time intervals. Using a similar approach, the absolute expression of salt responsive genes in different parts/anatomical structures of *Arabidopsis* plant was obtained. Subsequently, heatmaps were plotted using DISPLAYR online data representation software (https://app.displayr.com, accessed on 14 July 2021). Expression patterns of *NDL1* and its putative interactors at different developmental stages were analyzed using GENEVESTIGATOR [30] with default search filters in the compendium wide analysis of the software. The data were refined using the filter-only wild type genetic background.

Protein solubility of SRPIN upon overexpression was checked using the SOLpro database (http://scratch.proteomics.ics.uci.edu/, accessed on 3 April 2020).

### 2.2. Phenotypic Analysis under Salt Stress

Seeds of the different genotypes Col-0, *agb1-2*, *ndl1-2*, *NDL-GUS*, *NDL-GUS agb1-2,* and *rgs1-2* used in the present study were those from our previous studies [17]. For phenotypic analysis, seeds were surface sterilized and plated on half- MS agar medium. For the salt stress treatment, seeds were plated on half- MS agar plates supplemented with different concentration of NaCl (0, 125 mM and 150 mM). After two days of stratification in dark at 4 °C, percentage germination was determined. Seeds were grown up to 21 days in a growth room with photoperiod 16 h light/8 h dark, temperature at 22 °C, and light intensity of 100 μmol m^−2^ s^−1^. On day 15–21, all the seedlings were observed for changes in rosette diameter and chlorophyll bleaching compared to no treatment control. ImageJ software (available on https://imagej.nih.gov/ij/, downloaded on 20 January 2020) was used to calculate the rosette diameter.

### 2.3. Cloning

Coding DNA sequences (CDSs) of the selected putative interactors were amplified using a gene specific primer set (Table 1). Amplified fragments were ligated into a pENTR/D-TOPO entry vector (Invitrogen) at 16 °C for 1 h, as recommended by the manufacturer. Aliquots of the reaction mixture were transformed in to electro-competent DH5α cells. Plasmid DNA isolated from colonies were tested for respective genes by PCRs followed by cloning in to pACTGW-attR with Gal4 activation domain using LR clonase reaction (Invitrogen) at 25 °C, overnight, as recommended by the manufacturer. *NDL1* CDS was already available in pAS-attR vector with Gal4 DNA-binding domain from our previous study [17].

### 2.4. Yeast Complementation Assay

Yeast complementation was performed using a Gal-4 based two-hybrid system. CDS of SRPIN were cloned in-frame with a Gal4 activation domain into pACTGW-attR vector. CDS of *NDL1* with Gal4 DNA-binding domain in pAS-attR vector was already available to us from previous experiments. Constructs of *NDL1*-pAS-attR and SRPIN, namely *ANNAT1*, *SLT1*, and, *IDH-V*, were then co-transformed into the yeast strain AH109. Transformants were plated on to single drop-out (SD/-Leu for pACTGW-attR); (SD/-Trp for pAS-attR) and double drop-out (SD/-Leu/-Trp-for double transformants) media.

Yeast two-hybrid interactions of clones were further confirmed by colony lift colorimetric assay using 5-bromo-4-chloro-3-indolyl-β-d-galactopyranoside (X-gal) as substrate [31]. Briefly, transformants from the master plate were transferred to nitrocellulose filter by overlaying the membrane on top of the colonies. The nitrocellulose filter was lifted and frozen into liquid nitrogen, thawed at room temperature to lyze yeast cells, and placed in a Petri dish containing a disk of Whatman paper wetted with 2 mL Z-Buffer (60 mM NA_2_HPO_4_, 40 mM NaH_2_PO_4_, 100 mM KCl, 1 mM MgSO_4_ and 50 mM β-mercaptoethanol) and 0.6 mg/mL X-gal (β-galactosidase). The Petri dish was incubated for 14–16 h at 30 °C and blue colored colonies were scored positive for Y2H interaction. *NDL1* alone was used as a negative control for the tests. Each set of experiments was repeated thrice in order to confirm the results.

### 2.5. Yeast Salt Stress-Tolerance Growth Assay

Transformants obtained by Y2H complementation were used for stress tolerance assay. Yeast cells harbouring single vector were incubated in SD/-Trp (*NDL1*-pAS-attR) and SD/-Leu (*ANNAT1*-pAS-attR, *SLT1*-pAS-attR, *IDH-V*-pAS-attR) medium. However, yeast cells with two vectors (*NDL1*-pAS-attR and respective SRPIN) were incubated in SD/-Leu/-Trp medium containing 2% dextrose for 24 h at 30 °C. For stress treatments, exponentially grown yeast cultures having OD_600nm_ = 1.0 were used for preparing serial dilutions of 10^−1^, 10^−2^ and 10^−3^. Then, 10μL cell suspensions of each dilution were spotted on YPD medium with and without 1 M NaCl. Plates were incubated at 30 °C for 48 h and growth patterns were observed. For control, an equivalent number of AH109 cells were used.

## 3. Results

### 3.1. NDL1 Acts as Negative Regulator of Rosette Diameter and Chlorophyll Leaching and Work Downstream of AGB1 during Salt Stress

**Figure 1:**
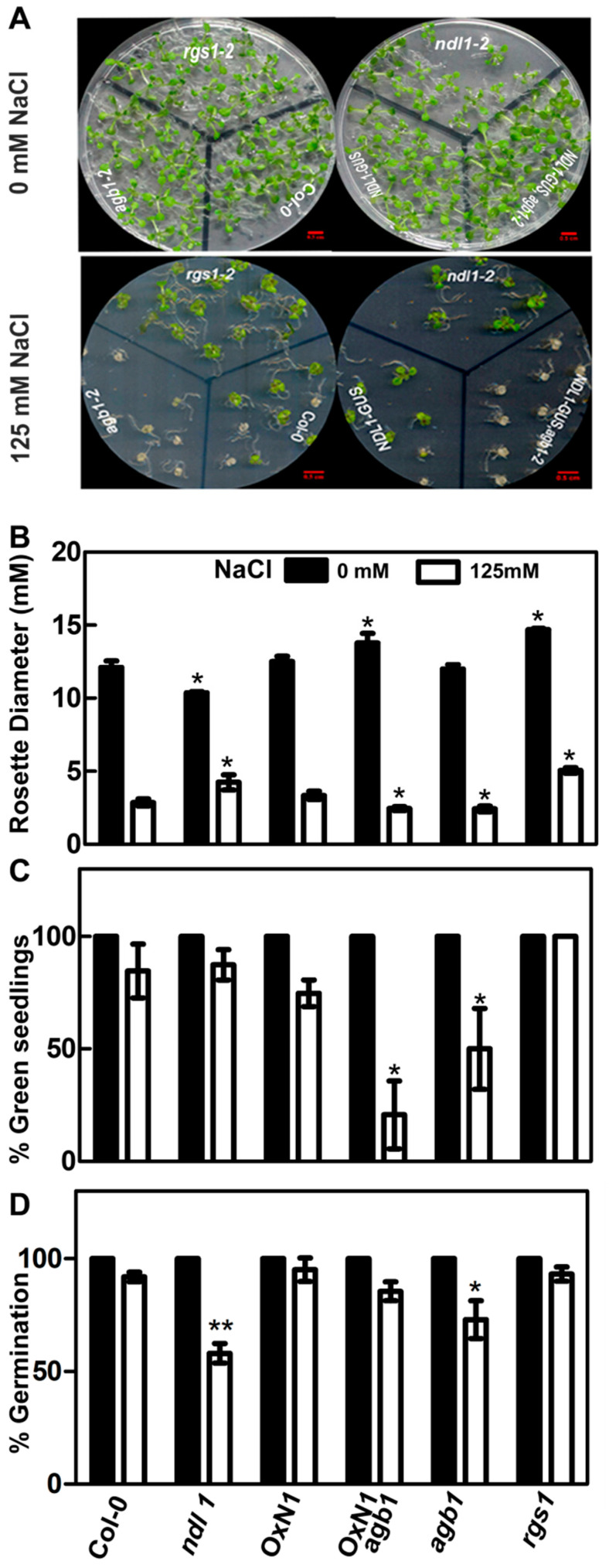
Phenotypic characterization of NDL1-AGB1 module response to salt stress. (**A**) Seedlings of Col-0; *rgs1-2*; *agb1-2*; *ndl1-2*; *NDL-GUS* (OXN1) and *NDL-GUSagb1-2* (OxN1agb1) genotypes were compared for their growth on 125 mM NaCl. Seeds were directly started on half-MS containing 125 mM NaCl and grown for 15 days followed by analysis. Scale bar = 0.5 cm. (**B**) Rosette diameter (mM) of the plants grown on 125 mM NaCl and plain MS were measured and compared using image J software. Error bars represent SD. Student’s *t*-test results are based on difference between Col-0 and indicated genotypes shown as asterisks: * *p* ≤ 0.05 and ** *p* ≤ 0.005. (**C**) Number of green seedlings were compared between different genotypes after 15 days after germination on salt stress and compared to no salt control. (**D**) Percentage rate of germination compared to no treatment control of Col-0 after 2 days of post germination.

We studied the in-planta functioning of this NDL1-AGB1 module in response to salt stress using various genetic backgrounds available for this module. We found that absence of *NDL1* (*ndl1-2*) which has established phenotype of slightly reduced rosette diameter, compared to Col-0, resulted in healthier growth of the 15-day-old seedlings on 125 mM NaCl in comparison to wild-type Col-0 control (Figure 1A). *ndl1-2* showed significantly larger rosette diameter (5.16 ± 1.2 mm), compared to Col-0 (3.3 ± 0.3 mm) a phenotype similar to *rgs1-2* and opposite compared to *agb1-2*. Similar to previously established phenotype of *agb1-2* [10], under salt-stress, we also found reduced *agb1-2* diameter (2.2 ± 0.1 mm) upon salt treatment as compared wild-type, Col-0 control (3.3 ± 0.3 mm). In the case of *NDL1* overexpression (*NDL1-GUS*) in Col-0 background, in both the cases, i.e., control and 125 mM NaCl, no significant difference was observed as compared to wild-type, Col-0 control. *NDL1* overexpression in *agb1-2* background (*NDL-GUSagb1-2*) under salt stress showed reduced rosette diameter (2.2 ± 0.1 mm) as compared to Col-0 plants, but almost equivalent to single mutant *agb1-2* (2.2 ± 0.1 mm) (Figure 1B). Since under control conditions there is no significant difference between *ndl1-2* and Col-0, but under salt stress *ndl1-2* shows healthier growth as compared to Col-0, this suggests that *NDL1* is a negative regulator of rosette diameter during salt stress response, a phenotype opposite to *agb1-2.*

*NDL-GUSagb1-2* when subjected to prolonged growth on salt showed more number of white/bleached or less green seedlings as compared to *agb1-2* and also the rate of bleaching was faster than *agb1-2* (Figure 1C). This demonstrates that the NDL1-AGB1 module is involved in regulating greening and whitening/bleaching of leaves.

### 3.2. NDL1 Acts as Positive Regulator of Germination during Salinity Stress and Work Downstream of AGB1

Reduction in germination rates of all the genotypes were observed during salt treatment experiments. In order to discover genotypic association with this phenotype, we calculated percentage germination rates under salt stress (125 mM NaCl) compared with no salt control condition. In the case of *ndl1-2*, germination showed hypersensitivity to salt treatment and lowest germination rate ~58% compared to wild-type, Col-0 (92%) control, and *agb1-2* (73%). On the other hand, *NDL-GUS* (OxN1) and *NDL-GUSagb1-2* (OxN1*agb1*) showed no significant difference compared to Col-0 germination rates, however they were more than *ndl1-2* and *agb1-2* (Figure 1D). This shows that *NDL1* might acts as a positive regulator during germination phase of the plant growth. *NDL-GUSagb1-2* showed nearly no decrease in germination percentage, comparable to *NDL-GUS*. This finding again supports the possibility that *AGB1* acts upstream of *NDL1*.

As expected, being less sensitive to salt treatment, *rgs1-2* showed germination percentages (93%) even better than the wild-type, Col-0 control.

**Figure 2:**
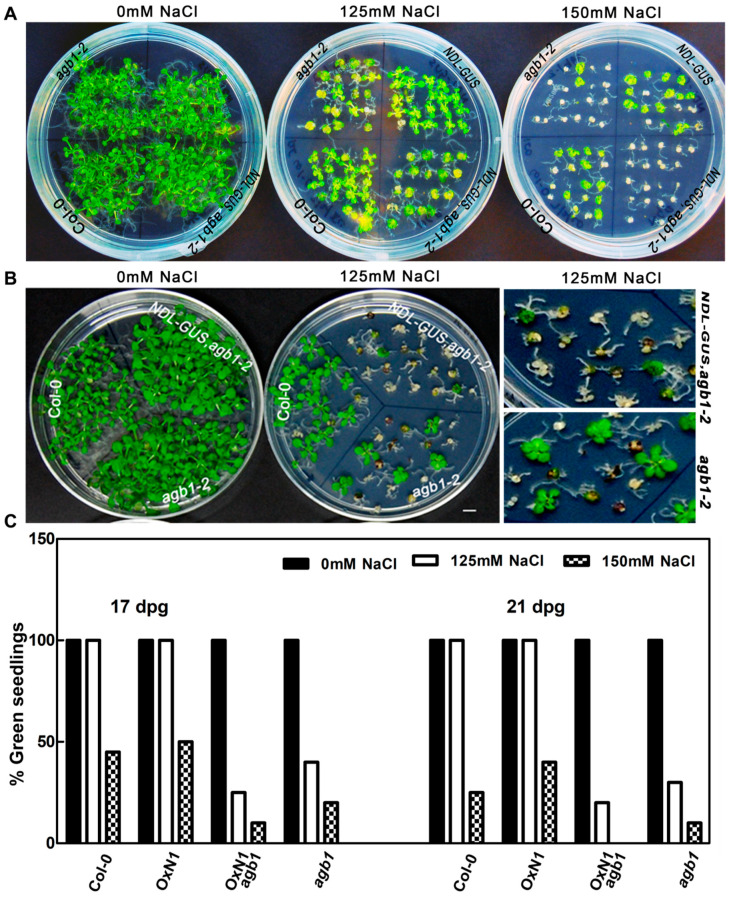
Phenotypic characterization of location of NDL1 in salt stress response. (**A**) Response of *NDL-GUS* (OXN1) and *NDLGUSagb1-2* (OxN1agb1) were compared to Col-0 and *agb1-2*. Seedlings were compared and tested for their growth on 125 mM and 150 mM NaCl on day 21. *NDL-GUSagb1-2* bleached faster than *agb1-2*. (**B**) Phenotypic characterization was also performed for shorter duration and concentration of NaCl (day 17 on 125 mM NaCl) in that case also *NDL-GUSagb1-2* showed faster rate or bleaching or whitening compared to *agb1-2* (enlarged view also provided). Scale bar for plates in A and B = 0.5 cm. (**C**) Graph showing number of green seedlings in genotypes mentioned above after 17 and 21 days post-germination at different concentration of NaCl. All the experiments were repeated more than four times with 15–20 seeds; a representative graph and image of one such experiment are shown.

In order to find location of *NDL1* in this pathway, native overexpression of *NDL-GUS* in the absence of *AGB1* (*NDL-GUSagb1-2*) was analyzed on NaCl over 17 days (125 mM NaCl) and 21 days (125 mM and 150 mM NaCl). We found that *NDL-GUSagb1-2* when subjected to either shorter or longer duration of growth on salt (for 17 or 21 days) showed an increased number of white/bleached seedlings as compared to *agb1-2*. Furthermore the rate of bleaching was faster than *agb1-2* (Figure 2A,B). This clearly demonstrates that *NDL1* functions downstream of *AGB1* in the pathway as the absence of *AGB1* results in the manifestation of *NDL1* function (more bleaching).

### 3.3. Salt Response Specific Components of NDL1 Interactome–Expression during Different Developmental Stages and Tissues

**Figure 3:**
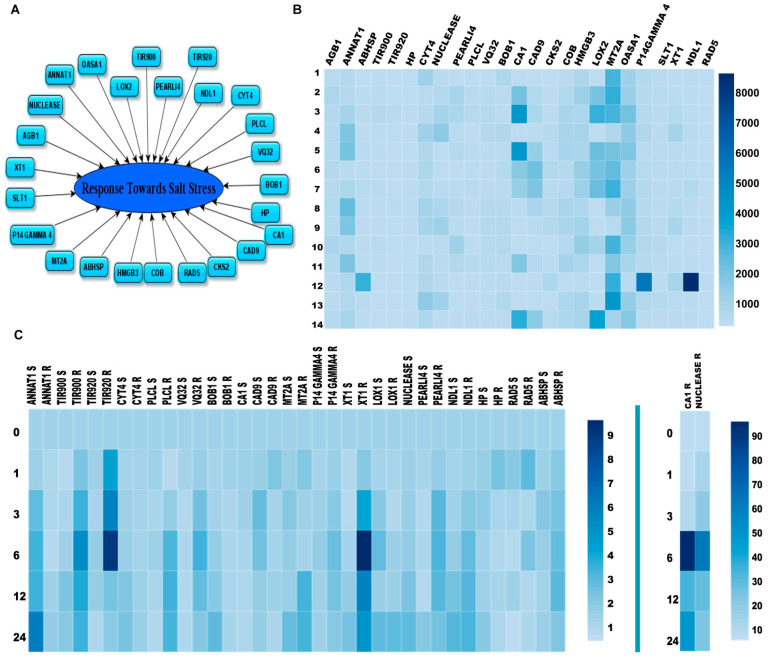
In-silico analysis of salt specific interactome of NDL1. (**A**) Putative interactors of NDL1 involved in salt stress responses from the interactome. (**B**) Heatmap showing in-silico expression analysis of 24 SRPIN by eFP browser (http://bar.utoronto.ca/efp2/Arabidopsis/Arabidopsis_eFPBrowser2.html, accessed on 20 March 2020) where the numbers are absolute expression values. The number on the left side of image represents; 1—Dry seed, 2—Cauline Leaf, 3—Cotyledon, 4—Root, 5—Entire Rosette After Transition to Flowering, 6—Flower Stage 9, 7—Flower Stage 15, 8—Hypocotyl, 9—Root, 10—Senescing Leaf, 11—Stem, 12—Mature Pollen, 13—Seeds Stage 10 w/o Siliques, 14—Vegetative Rosette. (**C**) In-silico microarray analysis heatmap showing expression of SRPIN in salt stress at different time intervals 0, 1, 3, 6, 12 and 24 h. Values obtained through ePF browser (Arabidopsis_eFPBrowser2.html, accessed on 20 March 2020) represent fold change in salt stress (*CA1* and *NUCLEASE* shown separately due to high range of expression values). Root and shoot in-silico microarray data of 15-day old seedlings of SRPIN after 150 mM NaCl treatment using eFP browser of TAIR (R and S added after gene names for expression in root and shoot respectively).

Our previous G-Protein interactome study have used NDL1 as a bait and discovered 62 interactions [19]. We performed in-silico analysis for each of the putative interacting partner of the NDL1 and discovered that out of 62 putative interactions, >50% of the interactors play a role in various kinds of stress responses. Detailed analysis of the NDL1 interactome showed that 24 of them are specifically involved during salt stress responses (Figure 3A and Table 2). We designate them salt stress related putative interactors of NDL1 (SRPIN) throughout the manuscript.

To identify stage and organ-specific expression of the genes at different stages of plant development; in-silico comparative expression analysis in different plant parts was carried out for the shortlisted candidate genes. The absolute expression values of SRPIN genes were obtained from eFP browser (http://bar.utoronto.ca/efp2/Arabidopsis/Arabidopsis_eFPBrowser2.html, accessed on 20 March 2020). SRPIN showed ubiquitous expression in different parts of the plant and across all the developmental stages but at different levels (Figure 3B and Supplementary Figure S1A) in different parts of plant. The expression pattern in different developmental stages was retrieved using GENEVESTIGATOR (https://genevestigator.com/, accessed on 22 March 2020) and showed ubiquitous and relatively higher expression of *MT2A* and *OASA1*. *MT2A* expression was found higher in most of the stages like developed rosette, young flower, developed flower, flower and silique, mature silique, and at senescence stage. OASA1 showed highest expression in stages like seedling, young rosette. However, both *MT2A* and *OASA1* showed high expression in germinated seeds and at the bolting stage. The overall expression levels of *MT2A* are high in all the parts of the plant, whereas the expression levels of *RAD5*, and *SLT1* remain low in all parts compared to all other SRPIN genes. *MT2A* showed high expression in dry seed, cauline leaf, flower (stages 9 and 15), senescence leaf, and siliques. *CA1* expression was high in cotyledon and, rosette after transition to flowering. *ANNAT1* showed higher levels in both above ground parts and root. *NDL1* along with *ABHSP*, *P14 GAMMA 4* showed highest expression in mature pollen; *LOX2* in vegetative rosette; *NUCLEASE* in roots. All the SRPIN showed differential expression pattern in different plant parts (Supplementary Figure S2). The pervasive expression of NDLs and SRPIN at all developmental stages and plant parts suggests their combinatorial role in plant growth and developmental processes. However, the differential expression levels across developmental stages also suggest distinct functions at different stages of development.

### 3.4. Detailed In-Silico Expression Analysis of SRPIN under Salt Stress

In order to identify potential interactions between SRPIN and the NDL-AGB1 module mediated salt stress signaling pathway, we carried out in-silico comparative analysis under salt stress using eFP browser. Interactors showing above a two-fold change in their expression at different time intervals under salt stress were shortlisted (Figure 3C and Supplementary Figure S1B). The interactors were found to be differentially expressed in shoot and root part under salt stress. Another point of interest was the expression of SRPIN in shoot and root part showed huge variation. For example, based on time intervals, Nuclease in roots showed maximum expression (seven-fold increase after1 h and 15-fold increase after 3 h treatment). *CA1* in roots showed a tremendous increase in expression after longer durations of treatment (98-fold after 6 h, 30-fold after 12 h and 46-fold increase after 24 h of treatment). Overall, on the basis of a greater than two-fold increase in expression, SRPIN genes like *CA1*, *NUCLEASE*, *XT1*, *TIR920*, and *ANNAT1* showed the highest levels of expression. A majority of genes show more than two-fold changes in either shoot or root. *ANNAT1* expression gradually increased over time in the shoot.

The expression of *TIR900*, *TIR920*, *PLCL*, *VQ32*, *P14GAMMA4*, *XT1*, *PEARLI*, *ABHSP*, and *NUCLEASE* showed increased expression in the root in the early hours of stress treatment followed by a steady decrease, and a similar trend was also seen in the case of *CAD9* in shoot. *CYT4*, *BOB1*, *MT2A*, *XT1*, *LOX2*, and *NUCLEASE* showed delayed upregulation in shoot. *CA1* and *LOX2* expression also increased with time and decrease gradually but in late hours their expression increased. *P14GAMMA4* expression increased in later hours, while the expression of *RAD5* was increased in early hours (before 6 h) of stress and decrease subsequently (after 6 h).

The high level of salt induced expression of *ANNAT1* in shoot and prominent literature on the role of *SLT1* in salt stress persuaded us to further analyse these two candidates along with *IDH-V*, which is part of NDL1 interactome and also showed response towards various other stresses, for further functional analyses.

### 3.5. Complementation and Stress Assays in Yeast to Confirm In Vivo Interactions between NDL1 and Select Candidates of SRPIN and Confirm Their Functional Dependency

**Figure 4:**
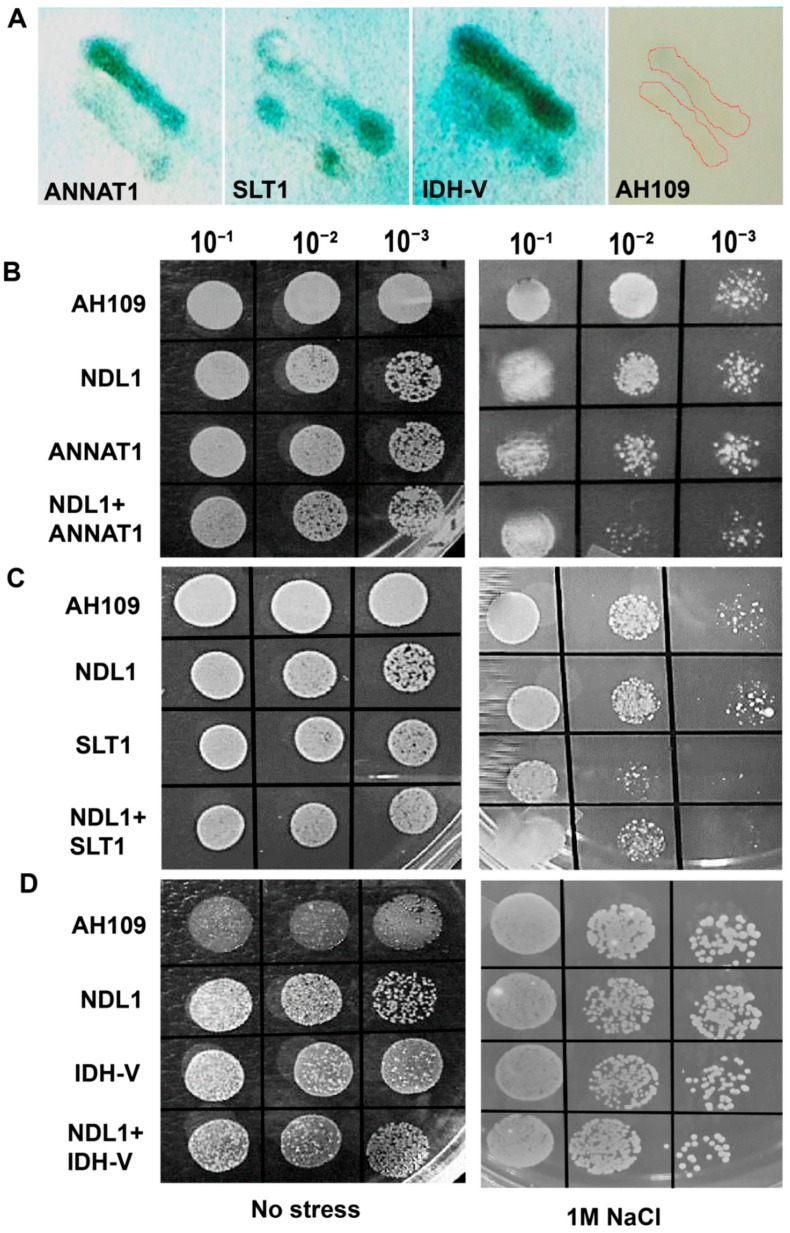
Analysis of salt specific interactome of NDL1 using Yeast. (**A**) Qualitative β-gal complementation assay to confirm in-vivo interaction of *NDL1* with their putative interactors using Colony Filter Lift Assay. β-gal complementation assay showing in-vivo interaction between NDL1 and select SRPIN candidates (ANNAT1, SLT1 and IDH-V). Images of filter lifts were taken after overnight incubation. No color was obtained for control having only single construct (*NDL1* alone). (**B**) Yeast cells growth was analyzed on 1 M NaCl, when both *NDL1* and *ANNAT1*, (**C**) *NDL1* and *SLT1* and, (**D**) *NDL1* and *IDH-V* were co-transformed together, cell growth in terms of number of the colonies formed was analyzed. Results shown are representative of yeast growth assay performed five times. Exponentially grown yeast cells were harvested and adjusted at OD600 = 1.0. Then, ten-μL of serially diluted culture were spotted on YPD plates with and without 1 M NaCl. Plates were incubated at 30 °C and growth was analyzed after 2 days.

Selected candidates of SRPIN from the previously established G-protein interactome were chosen. In-silico analysis about the solubility of the selected proteins was performed using SOLpro (http://scratch.proteomics.ics.uci.edu, accessed on 3 April 2020). ANNAT1 and SLT1 shown to be soluble with the probability of 0.63 and 0.79 respectively, while, IDH-V was shown to be insoluble with the probability of 0.61. To confirm the interactions between NDL1 and the selected SRPIN candidates (ANNAT1, SLT1, and IDH-V), all three members and *NDL1*-CDS were recombined in Y2H gateway vectors. Plasmids were co-transformed in to AH109 yeast cells and transformants were selected based on nutritional selection marker present on vectors. We further validated the findings by colony-lift filter assay to determine β-galactosidase (Lac Z) activity (Figure 4A). Blue colored colonies were scored for positive Y2H interaction. Tested colonies of all selected SRPIN, ANNAT1, SLT1, and IDH-V, with NDL1 showed blue staining on filter lift β-gal assay, suggesting positive interactions with NDL1. No color was detected in NDL1 alone control (Figure 4A).

To investigate the function of SRPIN candidates-ANNAT1, SLT1, IDH-V and that of NDL1 during abiotic stress responses, yeast growth assay was scored. *NDL1* and SRPIN were individually and co-transformed in to AH109, followed by the growth analysis. *NDL1* transformed along with *ANNAT1* showed reduced growth on 1 M NaCl compared to *NDL1* and *ANNAT1* alone, suggesting NDL1 and ANNAT1 interaction cancel each other’s effect during growth on salt stress (dil-10^−2^, Figure 4B). *NDL1* transformed yeast cells show better growth compared to *ANNAT1* alone.

In contrast, yeast co-transformation with *NDL1* and *SLT1* resulted in increased growth on 1 M NaCl (dil-10^−2^, Figure 4C) compared to *SLT1* alone but marginally reduced compared to NDL1 alone. Concomitantly, yeast cells show reduced growth with *SLT1* alone compared to *NDL1* alone. This suggests that *SLT1* down-regulates *NDL1* in salt stress pathway in yeast. In case of *NDL1* and *IDH-V* co-transformation growth marginally improved on 1 M NaCl (dil-10^−2^, Figure 4D). Individually transformed *NDL1* and *IDH-V* have comparable growth effects, suggesting that both function independently during stress responses.

## 4. Discussion

Shoot growth attenuation and leaf senescence are well documented phenomena in plants under salt stress. The rosette diameter of plants such as *Arabidopsis* tends to shrink under salt stress when compared with growth under no salt stress. High sodium is deleterious to most of the organisms, but halophytes, as opposed to glycophytes, can sequester Na^+^ inside vacuoles and exude it as a common mechanism of salt stress tolerance [38]. Being a glycophyte, at moderate salt concentration (e.g., 50 mM) growth of *Arabidopsis* is arrested. At salt concentrations of 100 mM, salt stress defects are observed in *Arabidopsis*, allowing investigators to discover stress phenotypes during plant growth and development and use it as a model. The role of G-protein components during salt stress has been already established, showing that under salt stress, loss of *RGS1* (*rgs1-2*) results in better growth of the shoot, while the loss of *AGB1* (*agb1-2*) results in chlorophyll leaching and rosette size reduction [10]. Small and chlorotic phenotype of different subunits of G-protein (*AGB1*, triple *XLG* and triple *AGG* null mutants) after salt stress treatments showed the involvement of G-protein during salt stress signaling [10,11,12]. Colaneri et al. (2014) hypothesized a role for G-protein during recovery phase after plants encounter with salt stress [10]. The accumulation of Na^+^ in both root and shoots of the *agb1-2* mutant indicates that AGB1 not only regulates Na^+^ flux in roots, but also regulates its translocation from roots to shoots [11,39]. In contrast to the *agb1-2* mutant, *rgs1* and *gpa1* mutants showed less chlorosis and larger rosette area compared to Col-0 after NaCl treatment [10]. Regulation of salt stress via G-proteins is very well studied in rice and maize too. Null mutant analysis of *Gα* subunit in rice and maize attenuates leaf senescence, chlorophyll degradation and electrolyte leakage [13]. Improved salt tolerance was detected in the overexpressing line of *RGG1* in rice [14]. NDL1 is an established interacting partner of AGB1 andAGG1/2, and this module functions in abiotic stress responses as inferred from expression patterns studies [40].

In our study, in-silico expression analysis of *NDL1* and SRPIN revealed specificity in the expression pattern at different development stages and in different anatomical parts, as well as differential expression patterns during the salt stress. The change in expression levels *NDL1* and SRPIN with time indicates that the expression was affected by salt conditions and their plausible role in the management of the same. Based on these in-silico findings, we hypothesize here that the NDL1-AGB1 module and these SRPIN work together during salt stress responses via G-Protein mediated signaling.

NDL1 is an interacting partner of AGB1 and the C4 domain of RGS1 subunit in *Arabidopsis* [17]. Animal (mouse) homolog of NDRG1 had been shown to interact with *Arabidopsis* AGB1/AGG1, AGB1/AGG2, and C-terminal domain of RGS1, suggesting that the interaction is evolutionarily conserved [17]. The G-Protein interactome was established in year 2011 mainly using yeast two hybrid followed by in-planta interaction methods. Numerous interactions (544) with 433 proteins were established in the G protein interactome. With NDL1 as bait, 62 interactions were discovered [19]. Detailed in-silico analysis for each of the putative interactors showed that ~73% of the interactors are involved in various biotic and abiotic stress responses [20]. Previous studies by different research groups show that many of these interactors are directly involved in various stress responses. O-Acetyleserine (THIOL) Lyase (OAS-TL) isoform A1 (OASA1) showed increased stress tolerance in response to cadmium [41].

*NDL1* potentially plays a role in stress-mediated microtubule organization [42]. Low water stress treatment leads to positive correlation of *NDL1* expression to the projected rosette area and increased rate of transpiration. Thus, *NDL1* was proposed as a biomarker for response to low water stress treatment [24]. Out of 73% putative interactors that play a role in stress responses, 24 are involved during salt stress responses and other abiotic stresses. Mutant analyses of *NDL1* showed increased rosette area and healthier plants in comparison to wild-type, under salt stress.

We hypothesize that *NDL1* works downstream of *AGB1* during salt stress responses. Furthermore, NDL1 interacts with putative downstream players to regulate signaling during salt stress. Our study identifies a dual role for NDL1, wherein it acts negatively and positively during salinity stress. This dual role depends upon the developmental stage of the plant growth. Our study identifies members of SRPIN (ANNAT and SLT1) that potentially interact with NDL1 in different organs to impose its dichotomous role. Absence of AGB1 is deleterious for plant growth in the presence of NaCl, but in contrast, loss of function of *NDL1* results in better growth, indicating negative role of *NDL1*. Plants overexpressing *NDL1* show the same phenotype as Col-0 plants. This could be because *AGB1* negatively regulates *NDL1*. As we previously reported, in the case of root apical meristems, *AGB1* presence is necessary to regulate steady state protein levels of NDL1 [17]. Similarly, during salt stress response, AGB1 regulates the negative regulator, i.e., NDL1. Plants with *NDL-GUSagb1-2* genotype show more bleaching than *agb1-2* plants. We speculate that in this case (a) *AGB1* is not present to stop *NDL1*, while (b) *NDL1* is expressed more. Both these possibilities act additively and result in excessive chlorophyll bleaching. Our results therefore suggest that *NDL1* acts downstream of *AGB1* in salt stress signaling.

Seed germination is regulated by different kind of signals and pathways that act both positively and negatively. It is mainly controlled by antagonism between gibberllic acid (GA) and abscisic acid (ABA). G-protein signaling is used by Arabidopsis to regulate hormonal control of seed germination [43]. Previous studies have shown indirect evidence of involvement of the GPA1 subunit of G-protein to regulate gibberellic acid and brassinosteriod signal transduction [44]. Plants lacking *AGB1* and *GPA1* subunits show hypersensitivity towards ABA [45]. *ndl1-2* shows significant reduction in percentage germination, suggesting that *NDL1* might act as a positive regulator during the germination phase of the plant.

Previously, *ANNAT1* mutants (*annat1*) in *Arabidopsis* were reported to show better tolerance towards salt stress [46] suggestive of its negative regulatory role. In the present study, reduced growth of the transformed yeast cells with *NDL1* along with *ANNAT1* on 1 M NaCl compared to their individual controls, suggests the negative effect of their interaction during salt stress. This underscores the importance of distinct effects of NDL1 and ANNAT1 interaction. Here, putative interactor IDH-V showed no difference in yeast cell growth with and without *NDL1*.

**Figure 5:**
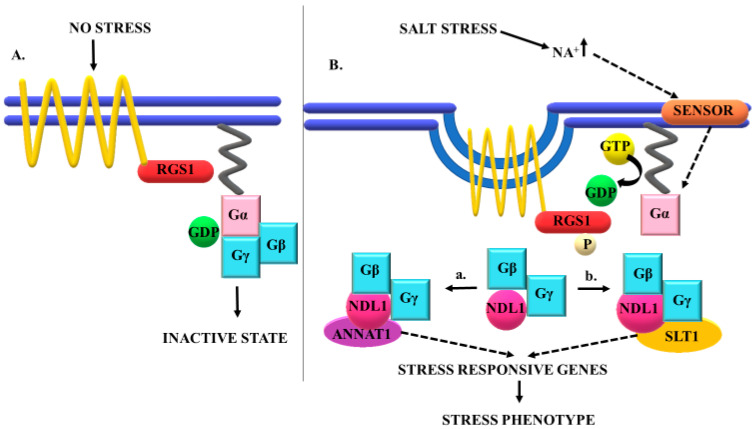
Proposed working model for G-protein meditated salt stress tolerance via NDL1; (**A**) no stress condition, ionic balance is maintained, G-protein is at inactive state. (**B**) Salt stress leads to activation of G-protein signaling, internalization of RGS1 takes place, exchange of GDP to GTP leads to activation of Gα subunit, followed by dissociation of Gβγ dimer. NDL1 interacts with Gβγ dimer and along with its putative interactors (a-ANNEXIN) or (b-SLT1) directly or indirectly leads to the activation of salt responsive genes and finally salt stress response.

Earlier, structural analysis of SLT1 had reported presence of an auto-inhibitory domain in the N-terminus of SLT1 that plays role in salt stress tolerance by modulating ions homeostasis [37]. Our results show reduced yeast growth in *NDL1* and *SLT1* compared to *NDL1* alone under salt stress, suggesting inhibitory effect of *SLT1* on *NDL1*. It is possible that in yeast, *SLT1* down-regulates *NDL1* in the salt stress pathway. Previous studies show that N-terminal truncated, but not full length, SLT1 protein mediates functional complementation of salt sensitive calcineurin-deficient yeast mutant [37]. Here, we show that, in the presence of NDL1 and SLT1 yeast grows better as compared to SLT1 alone under salt stress. We therefore speculate that NDL1 interacts with the N-terminal domain of SLT1 and inhibits its function. Thus, NDL1 and SLT1 may form an auto-inhibitory loop in salt-stress dependent signaling. NDL1 transformed yeast did not show any change in growth during salt stress, suggesting its function in context of interactions (SRPIN). We therefore hypothesize that, during salt-stress signaling, NDL1 interacts with downstream effectors and regulates the stress pathway, either positively or negatively, in a context-dependent manner. What then is the role of NDL1 in the context of upstream interactors? Previous studies have shown that NDL1 interacts with AGB1 and RGS1. In turn, AGB1 and RGS1 receive signaling from G-protein receptors. It is plausible that, during salt stress, G-proteins activate AGB1 and RGS1, which convey the signal to NDL1. NDL1 then interacts with SRPIN candidates, such ANNAT1 and SLT1, in a context-dependent manner to regulate stress response. Our study therefore supports NDL1 as an integrator of salt-stress pathways that determines outcome of salt stress response contextually. We have summarized the proposed role NDL1 in Figure 5. Further studies that decipher protein–protein interactions between NDL1 and SRPIN members will validate our working model.

## Figures and Tables

**Table 1:** Coding DNA Sequence (CDS) of the selected putative interactors were amplified using gene specific primer set.

S.No.	Gene Name	Gene ID	Primers (5′ to 3′)
1.	*ANNAT1*	AT1G35720	Forward CACCATGGCGACTCTTAAGGT
			Reverse AGCATCATCTTCACC GAGAA
2.	*IDH-V*	AT5G03290	Forward CACCATGACCATGGCAGCAAA
			Reverse GAGATGATCACAGATTGCCTTTG
3.	*SLT1*	AT2G37570	Forward CACCATGGAGAATCATCATCCTTCT
			Reverse TTAAGTCAGCATAAGATCGTTTCC

**Table 2:** NDL1 interactome showed twenty-four interactors specifically involved during salt stress responses. We refer to them as salt stress related putative interactors of NDL1 (SRPIN).

S.No.	Genes	Based on Online Available Sources	In-Silico above Two-Fold	References
1	*AGB1*	√		[27]
2	*ANNAT1*	√	√	[32]
3	*ABHSP*		√	
4	*TIR900*		√	
5	*TIR920*		√	
6	*HP*		√	
7	*CYT4*		√	
8	*NUCLEASE*		√	
9	*PEARLI4*		√	
10	*PLCL*		√	
11	*VQ32*		√	
12	*BOB1*		√	
13	*CA1*		√	
14	*CAD9*		√	
15	*CKS2*			unpublished
16	*COB*	√		[33]
17	*HMGB3*	√		[34]
18	*LOX2*		√	
19	*MT2A*		√	
20	*OASA1*	√	√	[35]
21	*P14 GAMMA 4*	√	√	[36]
22	*SLT1*	√	√	[37]
23	*XT1*		√	
24	*RAD5*		√	

## Data Availability

The in-silico data presented in this study are openly available in the the BioGIRD at doi:10.1093/nar/gkj109 [28], eFP Browser at doi:10.1371/journal.pone.0000718 [29] and Genevestigator at doi:10.1155/2008/420747 [30].

## Supplementary Materials

The following are available online at https://www.mdpi.com/article/10.3390/cells10092261/s1, Figure S1: In-silico analysis of Salt specific interactome of NDL1. (A). In-silico expression analysis of SRPIN by eFP browser in different organs and developmental stages of plant growth. (B). In-silico microarray analysis showing expression of SRPIN under salt stress. Values obtained through ePF browser represent fold change in salt stress. Root and shoot in-silico microarray data of 15 day old seedlings of SRPIN after 150 mM NaCl treatment using electronic fluorescent pictograph (eFP) browser of TAIR. Figure S2: In-silico expression analysis of SRPIN in different development stages using GENEVESTIGATOR (left to right): germinated seeds, seedling, young rosette, developed rosette, bolting stage, young flower, developed flower, flower and siliques, mature siliques and senescence (refer to Figure S1A for gene names).
