# Supplementary material for: Molecular Characterization of NDL1-AGB1 Mediated Salt Stress Signaling: Further Exploration of the Role of NDL1 Interacting Partners
Source: Cells. 2021 Aug 31;10(9):2261. doi: 10.3390/cells10092261 (PMC8472134; doi:10.3390/cells10092261)
Supplement: Supplementary file 1 [file cells-10-02261-s001.zip › cells-1283460-supplementary.pdf]

A.

		AGB1	ANNAT1	ABHSF	TIR900	TIR920	HP	CYT4	NUCLEASE	PEARLI4	PLCL	VQ32	BOB1	CA1	CAD9	CKS2	COB	HMGB3	LOX2	MT2A	OASA1	P14 GAMMA 4	SLT1	XT1	NDL1	RAD5
S.no.	Tissue	AT4G34460	AT1G35720	AT1G10740	AT1G72900	AT1G72920	AT4G16240	AT4G16500	AT4G29780	AT4G38550	AT4G38690	AT5G46780	AT5G53400	AT3G01500	AT4G39330	AT2G27970	AT5G60920	AT1G20696	AT3G45140	AT3G09390	AT4G14880	AT2G46500	AT2G37570	AT3G62720	AT5G56750	AT5G22750
1	Dry seed	192	307.42	10.26	57.01	0.2	26.86	825.72	127.39	14.09	0.49	5.32	408.85	3.52	11.34	15.23	135.62	496.68	5.15	2628.7	834.99	318.51	156.45	51.95	142.79	15.5
2	Cauline Leaf	440.25	277.13	69.1	136.06	32.96	36.04	145.91	294.63	654.51	122.65	182.15	148.61	1830.58	307.03	41.16	350.14	932.03	1713.5	2888.18	973.15	237.06	169.93	84.96	78.51	3.03
3	Cotyledon	288.78	702.1	118.06	273.99	185.5	46.91	257.74	731.75	492.83	229.44	198.88	58.2	4037.13	442.86	44.6	543.6	454.14	2808.65	2578.58	1843.31	241.46	101.86	269	79.63	2.01
4	Root	218.35	1597.48	50.31	81.81	28.71	39.96	877.18	1300.58	124.9	13.63	274.51	377.3	3.25	64.83	238.03	553.8	637.69	6.33	396.46	1250	370.31	104.73	711.13	115.58	5.58
5	Entire Rosette After Transition to Flowering	241.66	1772.96	95.83	66.76	14.76	28.18	393.5	25.45	278.15	102.73	74.98	142.2	3918.88	1083.75	108.16	621.46	533.46	2035.75	1832.2	1582.66	134.16	117.83	92.31	56.51	10.23
6	Flower Stage 9	297.34	140.11	70.34	11.36	6.96	40.73	501.68	35.69	15.14	37.48	12.98	265.25	1378.58	1935.43	312.95	208.48	1092.55	1779.71	2168.91	886.23	177.93	137.9	112.15	62.56	27.98
7	Flower Stage 15	255.36	479.03	159.38	107.75	14.88	65.76	333.61	118.2	107.98	111.13	50.93	155.96	1066.01	1610.25	102.23	219.94	890.68	2066.53	2754.35	902.25	332.28	145.29	113.83	319.21	7.38
8	Hypocotyl	268.96	2120.51	70.86	71.26	120.36	59.46	690.58	460.85	206.5	50.53	255.66	196.8	53.23	511.1	233.63	576.83	424.38	12.7	1012.51	1241.76	209.11	95.48	269.03	100.15	12.53
9	Root	256.71	1655.46	50.61	47.8	26.68	50.61	663.78	869.46	120.51	16.73	201.05	297.13	13.31	89.63	301.6	536.66	688.73	11.45	195.15	1291.26	331.45	108.63	563.08	98.11	16.18
10	Senescing Leaf	509.75	400.23	43.21	184.83	36.4	26.53	76.8	97.36	814.11	32.55	185.81	264.46	35.05	24.83	30.75	203.18	1107.43	723.15	2635.08	1021.03	306.13	199.86	27.65	48.3	1.38
11	Stem	333.5	1691.33	62.3	86.08	15.33	33.78	402.14	129.6	357.13	173.38	189.06	115.56	1554.41	211.81	70.56	718.83	471.31	7.88	1553.3	597.44	245.25	151.03	80.13	94.68	1.06
12	Mature Pollen	94.84	376.6	2831.94	10.81	1.26	29.8	377.05	120.78	2.76	2.93	68.31	125.91	6.61	40.71	409.11	26.73	81.75	7.13	2892.43	148.4	5423.03	59.65	645.91	8449.5	48.46
13	Seeds Stage 10 w/o Siliques	123.15	365.9	110.03	10.71	5.6	27.51	1271.45	792.48	18.61	1	73.45	338.85	4.89	25.51	26.41	478.16	614.73	18.56	4090.01	1119.41	361.85	247.86	144.61	100.78	9.58
14	Vegetative Rosette	197.66	652.98	76.06	17.03	6.38	42.55	429.65	47.01	44.3	108.65	54.18	150.18	2978.9	1303.41	162	425.3	664.8	3522.41	716.06	1418.11	114.31	130.35	91.38	96.08	21.43

B.

	Hrs	SHOOT	ROOT		Hrs	SHOOT	ROOT		Hrs	SHOOT	ROOT
ANNAT1	0	1	1	P14 GAMMA	0	1	1	BOB1	0	1	1
	1	1.11	0.66		1	0.8	1.2		1	0.79	0.98
	3	2.51	0.64		3	1.06	1.6		3	0.97	0.68
	6	3.09	0.41		6	1.38	2.36		6	1.34	0.67
	12	3.15	1.04		12	1.02	2.05		12	1.67	0.77
	24	5.86	1.41		24	1.09	1.65		24	2.6	0.83
TIR900	0	1	1	XT1	0	1	1	CA1	0	1	1
	1	0.39	1.98		1	0.53	1.71		1	1.1	0.15
	3	0.67	3.19		3	0.39	3.92		3	1.08	4.12
	6	1.03	5.11		6	0.96	9.53		6	0.75	98.22
	12	1.95	3.69		12	1.4	5.61		12	0.65	30.27
	24	1.67	3.18		24	2.1	4.7		24	0.61	45.82
TIR920	0	1	1	LOX2	0	1	1	CAD9	0	1	1
	1	1.07	4.09		1	0.86	0.8		1	1.22	1.85
	3	0.81	5.61		3	1.68	0.5		3	2.43	0.91
	6	1.34	8.97		6	2.52	1.17		6	2.18	0.86
	12	1.38	2.01		12	1.26	1.17		12	1.36	1.14
	24	0.66	1.53		24	2.56	2.56		24	1.35	0.68
CYT4	0	1	0.99	NUCLEASE	0	1	1	MT2A	0	1	1
	1	0.9	0.91		1	1.12	7.25		1	1.18	1.78
	3	1.11	0.89		3	1.27	14.92		3	1.72	1.09
	6	1.21	0.91		6	1.4	62.07		6	1.34	0.85
	12	1.86	1.13		12	2.03	24.7		12	1.26	3.08
	24	2.05	1.21		24	2.55	19.01		24	2.46	3.03
PLCL	0	1	1	PEARLI4	0	1	1	RAD5	0	1	1
	1	1.1	0.38		1	0.83	1.4		1	1.66	2.62
	3	0.73	1.38		3	0.44	2.78		3	0.81	0.6
	6	1.12	2.92		6	0.65	2.93		6	0.4	1.97
	12	0.72	3.11		12	0.46	2.76		12	1.03	0.54
	24	0.55	2.97		24	1.71	2.15		24	0.1	0.65
VQ32	0	1	1	HP	0	1	1	ABHSP	0	1	1
	1	0.93	1.27		1	1.25	2.06		1	1.1	1.63
	3	0.5	2.23		3	1.51	0.78		3	1.76	2.06
	6	0.64	3.15		6	0.99	0.53		6	1.26	2.43
	12	0.48	2.48		12	1.03	0.81		12	0.93	2.72
	24	0.57	1.75		24	1.67	0.65		24	1.18	1.86

Figure S1. In-silico analysis of Salt specific interactome of NDL1. (A). In-silico expression analysis of SRPIN by eFP browser in different organs and developmental stages of plant growth. (B). In-silico microarray analysis showing expression of SRPIN under salt stress. Values obtained through ePF browser represent fold change in salt stress. Root and shoot in-silico microarray data of 15 day old seedlings of SRPIN after 150mM NaCl treatment using electronic fluorescent pictograph (eFP) browser of TAIR.

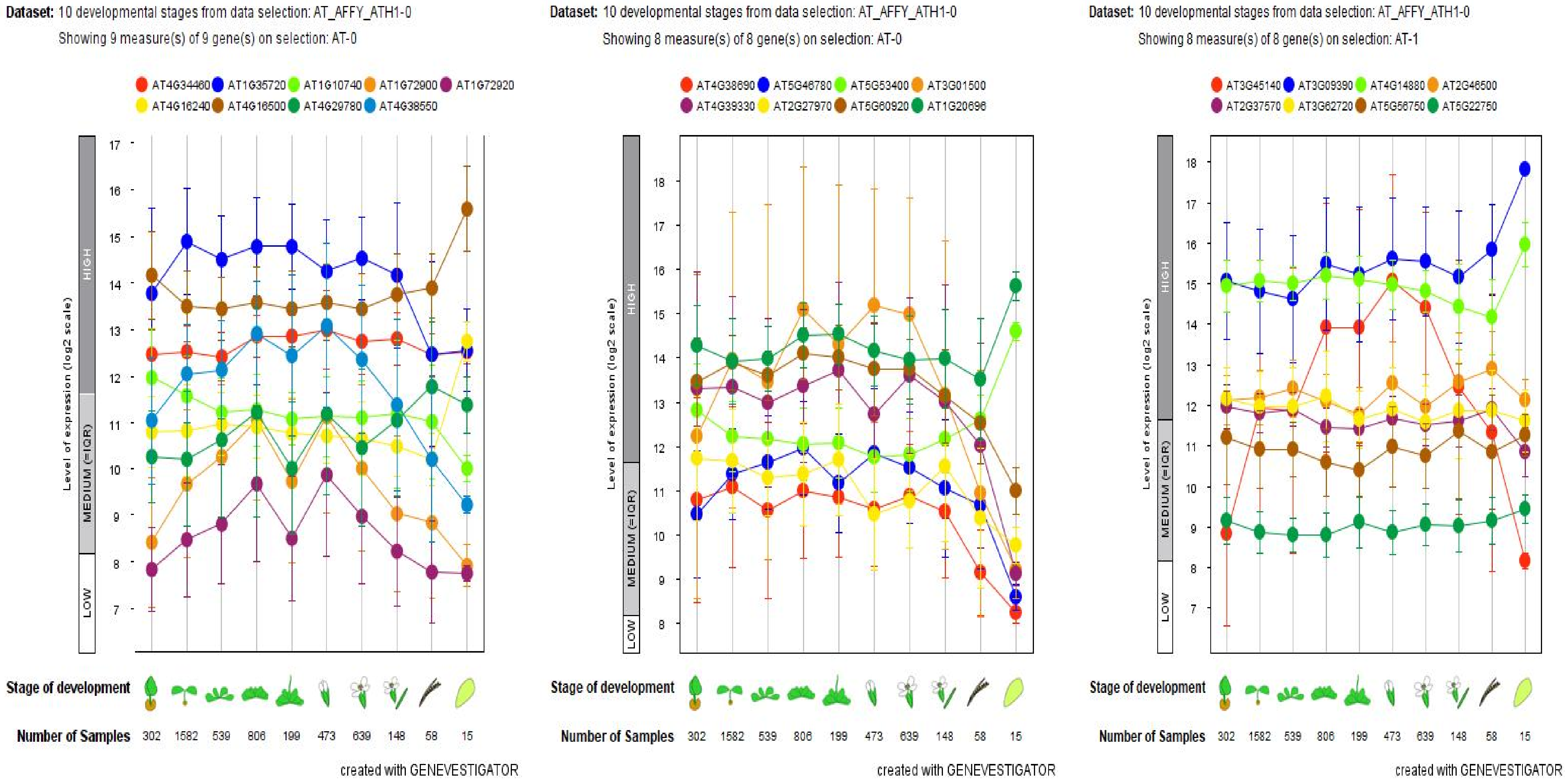

Figure S2. In-silico expression analysis of SRPIN in different development stages using GENEVESTIGATOR (left to right: germinated seeds, seedling, young rosette, developed rosette, bolting stage, young flower, developed flower, flower and siliques, mature siliques and senescence (refer to Figure S1A for gene names).
